# Chronic Diseases of Lifestyle: A Risk Assessment and Health Promotion Framework for a Rural and Urban Primary Health Care Setting in the Free State Province, South Africa

**DOI:** 10.3390/healthcare13010055

**Published:** 2024-12-31

**Authors:** Sanet van Zyl, Willem H. Kruger, Corinna M. Walsh

**Affiliations:** 1 Department of Basic Medical Sciences, Faculty of Health Sciences, University of the Free State, Bloemfontein 9301, South Africa; 2 Department of Community Health, Faculty of Health Sciences, University of the Free State, Bloemfontein 9301, South Africa; gngmwhk@ufs.ac.za; 3 Department of Nutrition and Dietetics, Faculty of Health Sciences, University of the Free State, Bloemfontein 9301, South Africa; walshcm@ufs.ac.za

**Keywords:** chronic diseases of lifestyle, primary health care, risk assessment, community-based education, CDL intervention programs

## Abstract

**Background:** Non-communicable diseases (NCDs) are the leading cause of global mortality. The WHO projects a rise in NCD-related deaths from 36 million in 2018 to 55 million by 2030, with developing countries being the most affected. Effective community-based primary health care (PHC) can reduce the burden of chronic diseases of lifestyle (CDLs). This study aimed to develop a risk assessment and health promotion framework to strengthen CDL prevention and control in Free State (FS) communities in South Africa. **Methods:** A convergent mixed-method design was used. Quantitative analysis identified CDL risk factors in rural and urban FS settings, while qualitative focus group discussions explored participants’ knowledge of CDLs and their experiences with program implementation. **Results:** Key findings highlighted differences in risk profiles, CDL training needs for PHC teams, patient education gaps, and curriculum development. Step 1 of the framework development identified differences and similarities in the CDL risk profiles of the study populations. Step 2 identified CDL training needs for PHC teams, patient educational needs, and CDL curriculum development needs. Step 3 revealed three main barriers: resource constraints, patient non-compliance, and the lack of supporting healthcare services. In Step 4, the six focus areas identified (steps 1–3) were used to develop strategies for implementing a tailored, community-based, patient-centred approach. **Conclusions:** The results provide valuable insights for improving PHC responses in resource-limited settings.

## 1. Introduction

Non-communicable diseases (NCDs), or chronic diseases, are the leading cause of death globally, accounting for 86% of premature deaths in low- and middle-income countries. South Africa faces multiple health challenges, including communicable diseases, maternal and perinatal issues, and an increasing burden of chronic conditions such as cardiovascular diseases (CVDs), diabetes, chronic respiratory diseases, and mental illness [1,2]. In South Africa (SA), NCDs account for 57.4% of deaths, surpassing those caused by communicable, maternal, perinatal, and nutritional diseases. Cardiovascular disease is the leading cause of death, followed by cancer, diabetes, and chronic respiratory disease [3,4]. In the FS province, NCDs cause 40.7% of deaths, with HIV/AIDS and tuberculosis being the next largest contributors [5]. In the rural Xhariep district, 58% of female and 46% of male deaths are due to NCDs, while the urban Mangaung district reports 59% of female and 49% of male deaths from NCDs [6].

Chronic diseases of lifestyle (CDLs) are NCDs that arise from long-term exposure to modifiable risk factors like unhealthy diets, tobacco use, and physical inactivity [7]. The high burden of CDLs places significant strain on primary health care (PHC) services in South Africa, as these facilities are the first point of contact for many patients. Hypertension and type 2 diabetes are among the top four conditions diagnosed at PHC facilities [8,9]. The focus has shifted from adult modifiable risk factors to broader determinants like socio-economic status and early life experiences, which perpetuate the cycle of CDL morbidity and mortality [10]. Subsequently, various strategies, including a life-course approach, have been suggested to acknowledge the influence of early life experiences and exposure to multiple risk factors throughout the lifespan that can impact adult health and mortality [11,12].

Current intervention strategies include integrated chronic care and management approaches guided by the WHO’s framework for preventing and controlling chronic diseases [13,14]. Despite the widespread adoption of integrated chronic care services at the primary care level in sub-Saharan Africa (SSA), several limiting factors still exist, including staff shortage and workload, physical resource constraints (especially in rural areas), and inadequate supply of medicine [13]. The WHO’s Innovative Care for Chronic Conditions (ICCC) Framework [15] emphasises evidence-based, cost-effective interventions to promote integrated, patient-centred care. Given South Africa’s escalating CDL burden, aligning these strategies with health system planning is crucial. This study aimed to develop a risk assessment and health promotion framework to strengthen existing efforts to prevent and control CDLs in rural and urban communities in the FS province.

## 2. Materials and Methods

### 2.1. Research Design and Setting

A convergent mixed-method design was used. The quantitative phase described CDL risk profiles in urban and rural populations in the FS [16], and qualitative focus group discussions explored participants’ knowledge of CDLs, including patients, PHC team members, and medical students, and their experiences with implementing CDL programs.

The quantitative phase used data from a cross-sectional, population-based study to assess the prevalence and risk factors for CDLs (type 2 diabetes, hypertension, CVDs) in rural and urban FS samples. Participants included adult household members aged 25 to 64 years from three rural communities in the Southern FS (575 participants) and a proportional cluster sample from urban communities within the University of the Free State service area (429 participants). Data included socio-demographic information, self-reported health details, medical examinations, and blood analyses for metabolic and inflammatory markers. Statistical analysis included determining CDL-associated risk factors for the rural and urban study populations. The detailed methodology applied in the quantitative phase of the study is described in Van Zyl et al. [16].

The qualitative phase involved focus group discussions with PHC staff, chronic disease patients, and medical students, exploring their experiences with CDL protocols and guidelines in PHC settings, the implementation of CDL programs, and the CDL curriculum. Audio-recorded data were transcribed, and thematic analysis was conducted using an inductive approach, including data coding, categorisation, and identifying emerging themes reflecting participants’ experiences [17]. The detailed methodology applied in the qualitative phase of the study is described in van Zyl et al. [18].

Approval was obtained from the Health Sciences Research Ethics Committee of the University of the Free State (UFS-HSD2017/1435), the Free State Department of Health, and local municipalities. All participants provided written informed consent.

### 2.2. Content and Purpose of the Framework

**Figure 1:**
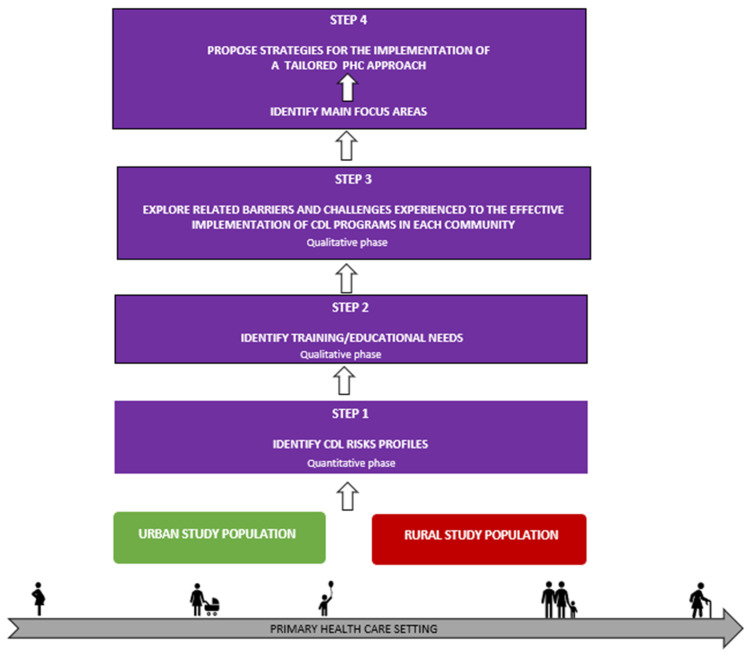
The four-step process followed to construct the CDL risk assessment and health promotion framework for the urban and rural study population.

The theoretical base (literature review), quantitative findings (CDL risk assessment for the study populations), and qualitative results (outcomes of focus group discussions with medical students, patients, and PHC teams) were integrated during the interpretative phase of the study. This was carried out through data triangulation to construct a framework and formulate recommendations to strengthen existing efforts for health promotion, CDL prevention, and control in the rural and urban communities in the FS. Figure 1 indicates the four-step process that was followed to construct the framework.

To compile the framework, this study integrated findings from the following: The WHO’s Innovative Care for Chronic Conditions (ICCC) Framework [15], the WHO Action Framework for the Prevention and Control of Chronic Diseases [14], the National Development Plan 2030 [19], Integrated Chronic Disease Management Model [20], Integrated Clinical Services Management Manual [21], and the Strategic Plan for the Prevention and Control of Non-Communicable Diseases, 2020–2025 [22].

## 3. Results

**Figure 2:**
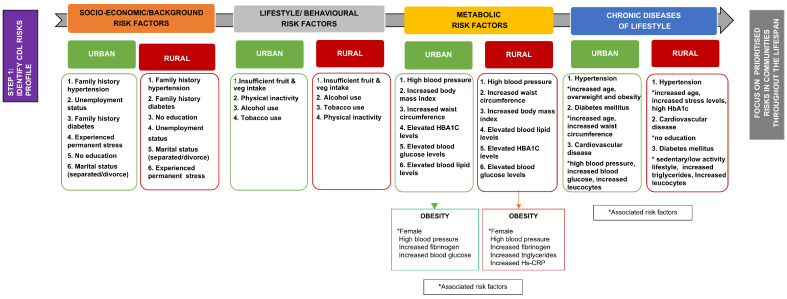
Prioritised risk and associated risk factors for obesity, diabetes, hypertension, and CVD in the rural and urban study population.

**Figure 3:**
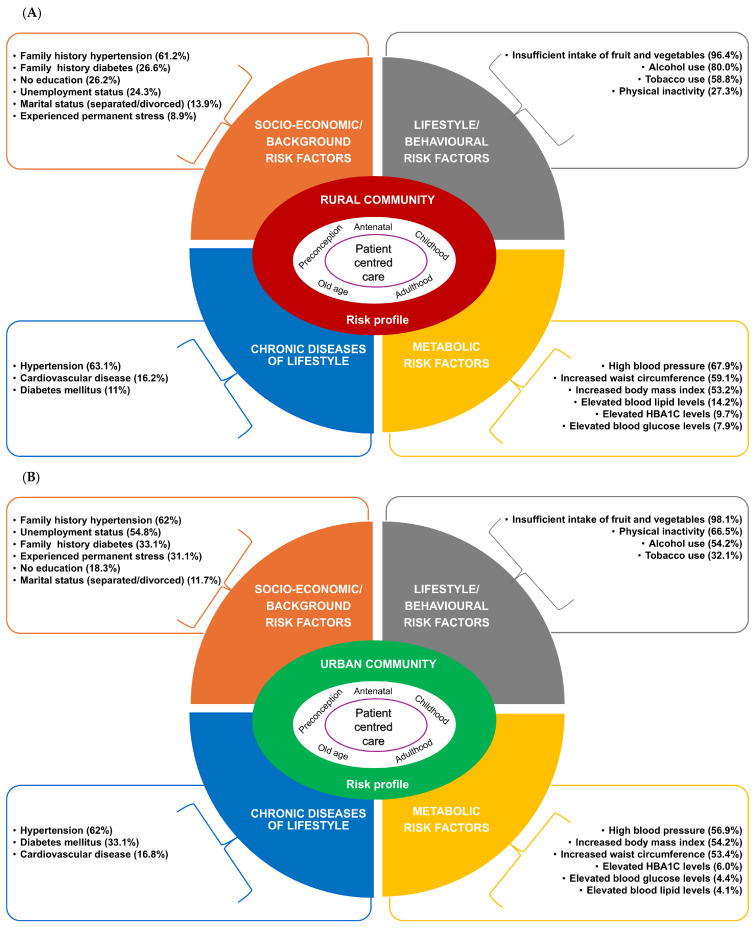
Step 1: Summary of prioritised socio-economic, behavioural, and metabolic determinants of CDL, CDL, and associated risks in (**A**) the rural study population and (**B**) the urban study population.

Step 1 of the framework development comprised constructing the CDL risk assessment profiles for the urban and rural study populations. Risk assessments allow the identification of several risk factors that deserve attention [23]. The prevalence of different socio-behavioural-metabolic risk factors associated with CDLs was summarised and prioritised to construct the CDL risk profiles for rural and urban study populations (Figure 2 and Figure 3).

The risk profiles highlighted similarities and differences in CDL risks between urban and rural populations. Van Zyl et al. [16] provided a detailed comparison of socio-behavioural-metabolic risk factors in these communities. The study found a high prevalence of modifiable risk factors such as low fruit and vegetable intake, physical inactivity, alcohol and tobacco use, obesity, high blood pressure, hyperglycaemia, and dyslipidaemia in both populations. The leading behavioural risk factor in both communities was insufficient fruit and vegetable intake, linked to socio-economic conditions, long distances to shops, and a shift toward a more “Westernised” diet low in fresh fruits and vegetables [24,25]. In both study communities, high blood pressure was among the top three risk factors (67.9% rural vs. 56.9% urban). More than half of the participants (53.2% rural and 54.2% urban) were either overweight or obese. In comparison, 59.1% of rural and 53.4% of urban participants had waist circumference measurements that exceeded the cut-off values. Significant differences (*p* < 0.01) between rural and urban samples were observed, including higher unemployment in urban than rural areas (54.8% vs. 24.3%, respectively). Additionally, three times more urban participants reported experiencing chronic stress, possibly linked to their unemployment status. Physical inactivity was significantly more prevalent in the urban (66.5%) than the rural (27.3%) study population (Figure 3).

**Figure 4:**
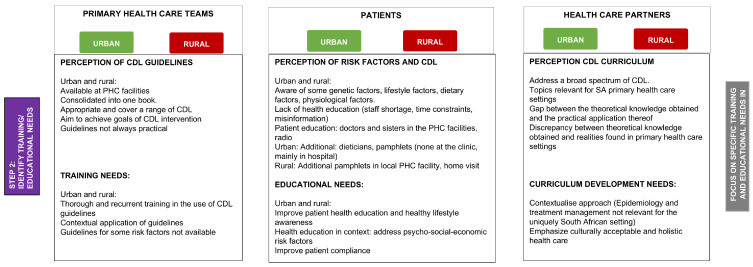
Step 2: Identified training needs for the effective implementation of CDL guidelines and protocols (PHC team), CDL educational needs (patients), and CDL curriculum development needs (medical students).

The risk profiles provided insight into the similarities and differences in CDL risks between the urban and rural study populations. Therefore, Step 2 of the framework development aimed to investigate participants’ knowledge and implementation of CDL guidelines and protocols (PHC team), knowledge of CDLs (patients’), and the application of CDL curriculum content in practice (medical students). A total of 61 participants participated in seven focus group discussions in the qualitative part of the study. Two focus groups were held with 22 medical students in total; 82% of the participants were female, compared to 18% males. A total of 39 participants, 20 patients with CDL, and 19 PHC team members participated in five focus group discussions, with more females (76.9%) than males (23.1%) participating in the focus group discussions. Community health workers, clinical associates, staff nurses, and professional sisters were among the PHC team members who participated in the study. *Perceived barriers and challenges to the implementation of Chronic Diseases of Lifestyle programs experienced by patients* and primary health care teams in an urban and rural primary health care setting in the FS and *medical students’ perceptions of the chronic diseases of lifestyle curriculum in health care settings in South Africa* has been described in detail in Van Zyl et al. [18]. The finding of Step 2 (summarised in Figure 4) revealed three main development areas: training needs (PHC team members), educational needs (patients), and curriculum development needs (health partners and medical students).

Both urban and rural PHC teams cited staff shortages and time constraints as barriers to CDL training and patient education (Appendix A, Table A1). They noted that existing guidelines do not fully account for patients’ socio-economic conditions. Additionally, patient non-compliance and lack of ownership of their conditions were seen as contributing to the high intra- and intergenerational CDL burden in these communities. This study revealed important patient educational needs (Figure 4). While patients with chronic conditions (hypertension, type 2 diabetes, and CVDs) were aware of genetic and lifestyle risk factors for CDLs, they lacked detailed knowledge and held misconceptions, such as believing hypertension is infectious and that drinking water or using cooked salt can prevent diabetes and hypertension reflected in the following statements “*I want to know the cause of hypertension; my mother and dad don’t have it. I am the only one who has it. I want to know who infected me.*” (Patient Urban No 6); “*The salt we eat must be cooked with the food; the raw salt is very dangerous.*” (Patient Urban No 8). Despite the high prevalence of overweight and obesity observed in both study groups (Figure 3), maintaining a healthy body weight was not mentioned as part of healthy lifestyle choices during focus group discussions with patients. Patients attributed the lack of health education to various factors, such as staff shortages and related time constraints, and the factors elaborated on below.

Regarding CDL education and healthy lifestyle information, urban and rural patients indicated that they receive health education from physicians during consultations (focused mainly on treatment management and not healthy lifestyle information), nursing staff in the clinic, radio, and pamphlets (rural). Patients (urban and rural) mentioned a lack of supporting health services (dietetics, physiotherapists). Urban participants revealed that these services were previously available but reported that no functional support groups for CDLs (hypertension, diabetes support groups) had been available for the past few years. Furthermore, urban participants stated they had not been visited at home by PHC team members, community health workers, or health promoters. Advertisements and health awareness days were also not cited as contributing to patients’ health knowledge. In the rural setting, the patients indicated that the clinic nursing staff mainly contributed to what they knew about CDLs. At the same time, home visits by PHC workers in rural areas were also cited as contributing to patients’ understanding of their disease. During focus group discussions with rural PHC team members, this was confirmed. However, participants proposed more effective community outreach programs to improve patient education. One participant highlighted: “*We need a professional nurse. She has to go with us, so if you have a problem, they (patients) can go straight to her*” (PHC worker Rural No 6). This study identified important patient health education needs in urban and rural study populations. Addressing the patients’ educational needs, improving the training needs of PHC teams, and applying health education and interventions in context can contribute to improved patient compliance in these communities.

Focus group discussions with medical students emphasised the importance of contextualising educational programs in these communities, focusing on affordable, culturally acceptable, and holistic healthcare strategies for CDLs. Students also identified CDL curriculum development needs; for example, certain CDL content needs to be incorporated or revisited at strategic points throughout the curriculum to result in an effective integrated curriculum preparing students for the changing landscape of health care. Furthermore, emphasising socio-cultural-behavioural-economic determinants throughout the CDL curriculum can enhance students’ socio-cultural sensitivity and understanding of challenges experienced in urban and rural communities. *Medical students’ perceptions of the chronic diseases of lifestyle curriculum in health care settings in the Free State, South Africa have* been described in detail in Van Zyl et al. [18].

**Figure 5:**
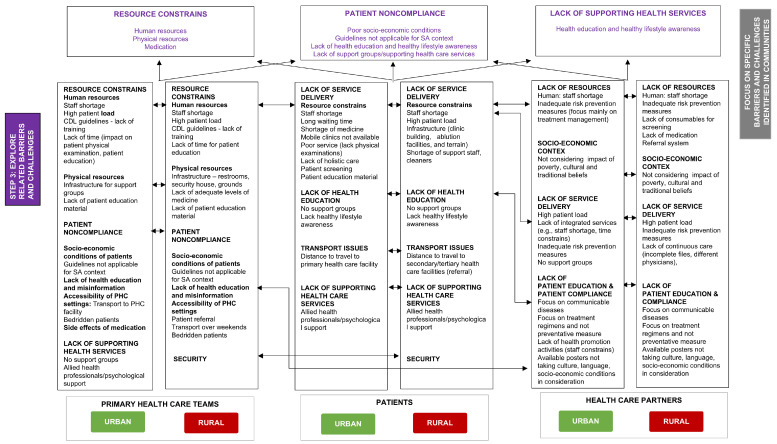
Step 3: Challenges and barriers faced to the effective implementation of CDL programs in the urban and rural study populations.

During Step 3, the integrated results obtained from focus group discussions with PHC team members, patients, and medical students identified resource constraints, patient non-compliance, and the lack of supporting health services as three significant barriers to the effective implementation of CDL programs in the urban and rural study populations (Figure 5). PHC team members indicated that the lack of human resources (staff shortages) contributed to the lack of time for proper physical examination of patients (urban), patient education (urban and rural), and training of staff members (urban and rural). In addition, the lack of physical resources—for example, the lack of patient education material (urban and rural), infrastructure for support groups (urban), and inadequate levels of medication (rural)—was mentioned as a contributing factor to the effective implementation of CDL educational, prevention, and intervention programs.

Medical students attributed the poor control of CDLs in the communities to a lack of human and physical resources. They confirmed observations of staff shortages (urban and rural), inadequate levels of medication (rural), and lack of patient education material (urban and rural). Furthermore, the lack of service delivery was attributed to high patient load (urban and rural), incomplete files, different physicians (rural), and patients’ socio-economic conditions (urban and rural). This is reflected in the following statements: “*Our system is very understaffed. We have a shortage of doctors and health staff*” (Medical student No. 6); “*Regarding the interventions, I think it is quite lacking due to not having the equipment, not having the manpower to see all those patients and serve the whole community*” (Medical student No. 7). In addition, students observed that the primary setting focuses mainly on communicable diseases and their treatment regimens, and that preventative measures or holistic care are not effectively implemented. In other South African studies [26], the emphasis on communicable diseases at the PHC level was also observed while observing deficiencies in CDL patient care.

Patient non-compliance was also identified as a significant barrier to the effective implementation of CDL programs. Both PHC teams and medical students confirmed that socio-economic factors, such as poverty, unemployment, food insecurity (urban and rural), accessibility to PHC settings (urban and rural), and drought (rural) made it difficult for patients to comply with and follow health education guidelines (such as following a healthy balanced diet) to reduce CDL risk. This is reflected in the following statements made by PHC workers: “*Some of the guidelines, I say they are impossible to implement due to the economics of our community*” (PHC worker Rural No 3). “*We used to advise the patients to have a small garden, but now it is very difficult to advise them because there is no water*” (PHC worker Rural No 2); “*Poverty is the problem; most of them cannot afford it*” (PHC worker Urban No 1). During community-based education (CBE) rotation, medical students also observed the socio-cultural and economic impact on health in these communities. They commented on the different aspects as follows: “*We should have been taught what food is available to the patients, what are the cultural norms*” (Medical student No. 4). “*It is very important that they customise the interventions for a South African setting*” (Medical student No. 9). “*We focus more to restrict salt intake, but what happens if you (refer to the patient) are depressed, suicidal, burnout, or if stress is causing your illnesses?*” (Medical student No. 12). The findings illustrate the limitation that socio-economic factors, including poverty as an important risk factor, placed on patients and households in these communities and their ability to comply with current preventative strategies, such as healthy lifestyle and behavioural approaches. Socio-cultural and economic factors restrict patients’ compliance and ability to implement necessary lifestyle changes [27]. However, related chronic psychological stress can also alter the physiological immune responses, contributing to CD morbidity and mortality [28] and the vicious cycle of CDLs [14]. In the qualitative phase of the study, patients confirmed that they were experiencing stress (reported in Step 1), and this was again confirmed in the qualitative phase (reported in Step 3), with patients in both communities confirming that experienced stress was related to socio-economic challenges (poverty, unemployment, drought): “*I do not have money, my children do not have shoes, they do not have water. The stress goes up.*” (Patient rural No. 7); “*Most of the people don’t work, don’t have a job, and they don’t have money, but when you come to the clinic, they refer you to the hospital, and you have to pay the taxi, but you don’t have money for transport*” (Patient rural No. 8). This illustrates the importance of following a patient-centred, contextualised approach as socio-cultural and economic factors experienced in a community may heavily impact patient compliance and the effectiveness of intervention programs.

The third identified barrier to effectively implementing CDL programs in the communities was the lack of supporting health services. Patients mentioned the lack of supporting health care services (e.g., dieticians, physiotherapists). Urban patients emphasised the need for active support group sessions focusing on healthy lifestyle coaching and psychosocial support to enhance patients’ self-care and ability to manage their chronic condition.

Step 3 revealed three main barriers: resource constraints, patient non-compliance, and the lack of supporting healthcare services to effectively implement CDL programs in urban and rural communities.

**Figure 6:**
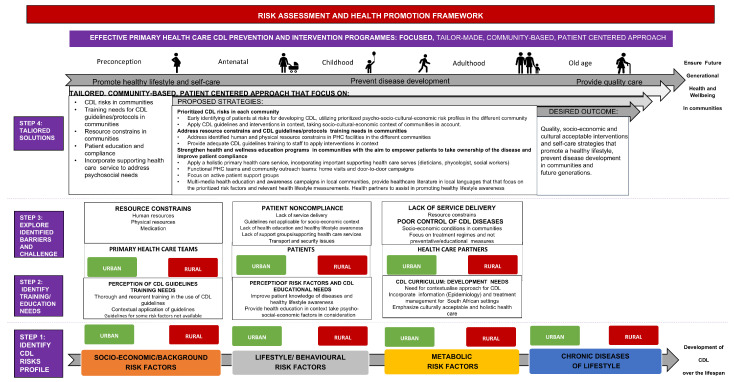
CDL risk assessment and health promotion framework for the urban and rural study population.

During the final step, Step 4, the six main focus areas (prioritised CDL risks in communities, training needs, resource constraints, patient education, supporting health care services, patient non-compliance) identified in steps 1–3 were used to develop a tailored community-based, patient-centred approach (Figure 6) to improve the effectiveness of existing CDL prevention and intervention programs in urban and rural communities and eventually improve the health and well-being of all living in the communities.

## 4. Discussion

With the growing burden of CDLs, preventive and long-term management strategies have become increasingly important. Many countries work towards the WHO’s global strategy of reorientation and strengthening health care towards high-quality, effective primary and community-based care [13,29,30]. Quality healthcare is underpinned by patient-centred care, also known as person-centred care, which provides health services and promotes health centred on people’s needs and preferences [29,30]. Furthermore, the WHO calls for the improved management of NCD, including interventions that focus on the risk factors for CDLs in the most cost-effective way to prevent and control the diseases in communities [1]. Patient-centred health services tailored to individual needs may provide better access, improved health literacy, increased patient participation in health care planning, and increased capacity to self-manage and control long-term health conditions, among other benefits [31].

This study confirms the high burden of CDLs in both urban and rural FS communities and emphasises the need for effective, targeted PHC prevention and intervention programs. The findings highlight the importance of a contextual understanding of CDLs’ multi-factorial aetiology (social, economic, cultural, psychological, behavioural, and metabolic determinants) and barriers and challenges experienced at the PHC level that drive the CDL disease process in these communities.

Based on the findings of this study, the researchers recommend the following strategies (summarised in Table 1) focused on a tailored community-based patient-centred PHC approach:

**Primary healthcare facilities and primary healthcare teams.**

- Target the multi-factorial aetiology of CDLs in the communities.
  ○ Implement intervention programs focusing on the prioritised CDL risks and the risk factors associated with CDLs (obesity, type 2 diabetes mellitus, hypertension, CVD) in each community (Figure 3) and optimise protocols to incorporate community-specific contexts.
  ○ Enhance the screening for CDLs and associated risk factors using essential point-of-care devices that can immediately identify critical biochemical markers (e.g., HbA1c, Hs-CRP, fibrinogen).
  ○ Apply CDL guidelines and interventions in context; for example, consider the socio-cultural context of communities while implementing protocols, guidelines, and interventions.
- Address the identified resource constraints and staff training needs to improve patients’ access to efficient and effective PHC services.
  ○ Address identified human and physical resource constraints in the different PHC facilities in the urban and rural communities.
  ○ Provide adequate and recurrent CDL guidelines training to enhance the ability of staff to apply interventions effectively.
- Apply a range of complementary, innovative, context-appropriate health education strategies that focus on the needs of CDL patients and address the challenges and barriers the patients have identified in the study. Strategies can include:
  ○ Effective health education and awareness campaigns implemented at local clinics that include, for example, multi-media approaches that consist of video recordings followed by live questions and answer sessions/health talks; assist patients in the use of new technologies, for instance, eHealth/healthy lifestyle applications [32]; display of multi-lingual educational posters and pamphlets (also see the section on Health partners below).
  ○ Active support group sessions that include CDL health literacy, healthy lifestyle coaching (focusing on implementing healthy lifestyle recommendations and patients’ ability to manage their condition), and psychosocial support that can contribute to patient compliance.
- Place more emphasis on the multidisciplinary team and the complementary roles of various health professionals involved in the holistic care of patients with CDLs. Apply a holistic PHC service by including essential supporting healthcare staff members (dieticians, phycologists, social workers, and healthy lifestyle coaches) as part of the community PHC team.

**Patients and community.**

- Implement various interactive health education strategies that focus on the prioritised CDL risks identified in each community to improve the effectiveness of health and wellness educational programs in communities, empowering patients to take ownership of their disease and improve patient compliance.
  ○ Functional PHC and community outreach teams that expand the integrated, holistic, patient-centred approach beyond the PHC facility into the community: home visits and door-to-door campaigns.
  ○ Active patient support groups outside the PHC facility (e.g., church groups) can effectively support health education efforts.
  ○ Investing in multi-media health education and awareness resources (e.g., pamphlets, posters) in local languages in the communities that focus on identifying prioritised risk factors and promoting associated healthy (anti-inflammatory) lifestyle interventions throughout the individual’s life course. This can be carried out in conjunction with health partners (e.g., health science partners). Distribute health information/educational material to local businesses, print media, and awareness campaigns. Provide the resources to teachers to enhance awareness of significant risk factors for CDL in schools.

**Health partners** (including other health professionals involved in training undergraduate medical students in the Faculty of Health Sciences).

- Incorporating the following essential components in community-based chronic disease learning modules for undergraduate MBChB programs:
  ○ Development of soft skills (emotional (EQ), social (SQ), and cultural (CQ) intelligence) from the foundation phase (pre-clinical phase). Integrating socio-cultural and emotional experiences as part of a student’s professional development (an e-portfolio) throughout the curriculum can assist students in developing socio-cultural and self-awareness.
  ○ Incorporate healthy lifestyle counselling skills development opportunities (training opportunities/webinars) into the pre-clinical CDL curriculum component. Involve students in developing healthy lifestyle information/educational material (using the above-obtained skills) that can be distributed in the communities. These activities can be assessed and incorporated into a student’s professional development (an e-portfolio).
  ○ Appropriate preparation of health professional students before CBE rotation, for example, the use of the WHO “Package of Essential Non-communicable (PEN) Disease Interventions for Primary Health Care” as part of pre-clinical educational courses [33].
- Work towards a national curriculum for CDLs, through a collaborative effort between educationalists and policymakers, to produce healthcare professionals that are sensitive to communities’ health, physical, mental, and social well-being needs and ultimately contribute to a healthy life for all.

The National Strategic Plan for the Prevention and Control of NCD’s aims to move South Africa closer to the United Nations Sustainable Development Goal 3.4 to reduce, *by one-third, premature mortality from NCDs through prevention and treatment* by 2030 and has therefore prioritised prevention and control of NCDs and promoting health and wellness across the lifespan [3,34]. Aligned with these goals, this study provides evidence-based, targeted and context-specific prevention and health promotion strategies that can strengthen existing efforts to reduce the heavy burden of CDLs in these communities.

The researchers recommend using prioritised CDL risk profiles to optimise current programs by applying context-specific protocols and guidelines and improving screening for identified risk factors. A range of innovative, context-specific health education strategies focused on prioritised CDL risks is also recommended to meet patient needs and address identified challenges.

A multidisciplinary team approach is critical, highlighting the complementary roles of dietitians, psychologists, social workers, and lifestyle coaches in providing holistic care for CDL patients. Strengthening the contribution of health partners, including professionals from various disciplines, through community-based chronic disease learning modules for medical students and fostering collaboration between educators and policymakers can produce healthcare professionals who are sensitive to the physical, mental, and social well-being of communities, ultimately contributing to better health outcomes for all South Africans.

### Limitations

The authors acknowledge the following limitations. Due to resource constraints, the study was limited to the FS province in central South Africa. Additionally, more females participated in the quantitative phase, likely because the study was conducted on weekdays when employed males were unavailable. The small sample size of 22 medical students, 19 PHC team members, and 20 patients with CDLs who participated in the focus group discussions may not fully represent the broader community. However, data collection continued until saturation was reached, and key sub-themes were consistently identified across all groups.

## 5. Conclusions

This study confirmed the high burden of CDLs and associated risk factors in urban and rural FS populations and barriers and challenges at the PHC level were highlighted, demonstrating a clear need for effective, focused PHC interventions to reduce the CDL burden. Based on the study’s findings, the researchers developed a risk assessment and health promotion framework and proposed a community-based, patient-centred PHC approach targeting six key areas: prioritised CDL risks, training needs, resource constraints, patient education, supporting healthcare services, and patient non-compliance. Therefore, the study’s findings can be valuable for planning, designing, and implementing focused community-based primary healthcare responses in resource-constrained areas.

## Figures and Tables

**Table 1:** Summary of the six focus areas identified and proposed strategies for implementing a tailored community-based patient-centred PHC approach in the urban and rural communities.

Main Focus Areas	Proposed Strategies for the Implementation of a Tailored, Community-Based Patient-Centred PHC Approach in the Urban and Rural Communities		
	PHC Facilities and PHC Teams	Patients and Community	Health Partners (MBChB Students)
1. Prioritised CDL Risks in Communities	Use identified prioritised psycho-socio-cultural-economic risk profiles, early screening toolkits, and essential point-of-care devices in the different communities for early identification of patients at risk.	Apply CDL guidelines and interventions in context—consider communities’ socio-cultural-economic context.	Curriculum development: Refresher courses before CBE rotation in the clinical phase that focuses on the prioritised CDL in urban and rural communities (for example, treatment management refresher courses, information on supporting health services, and different referral levels). Development of soft skills (emotional (EQ), social (SQ), and cultural (CQ) intelligence). Incorporate healthy lifestyle coaching skills development opportunities. Work towards a national curriculum for CDL.
2. Training Needs	Provide adequate CDL guidelines training programs to enhance the staff’s ability to apply interventions in context.	Focus on the prioritised risk identified in the communities during healthy lifestyle counselling sessions (patient support groups) and health education campaigns (school programs, home visits, and door-to-door).	
3. Resource Constraints	Address identified human and physical resource constraints in PHC facilities in the different communities.		Health partners develop educational material on healthy lifestyles as part of module-specific outcomes in the pre-clinical phase.
4. Patient Education	Investing in multi-media health education and awareness campaigns that promote healthy lifestyle choices throughout the individual’s life course.	Focus on the identified and prioritised CDL risks in each community to strengthen focused health and wellness educational programs in the communities. Provide healthcare literature in local languages that focus on the prioritised risk factors and relevant health lifestyle measurements.	Health partners to promote a healthy lifestyle during CBE rotations in the clinical phase (using various methods: educational material, health dialogue during support groups, home visits, and door-to-door campaigns).
5. Supporting Health Care Services	Supply essential supporting health care services (dieticians, psychologists, social workers, and healthy lifestyle coaches). Focus on active patient support groups.	Functional PHC/community outreach teams that expand the focused, tailored, patient-centred approach beyond the PHC facility into the community: home visits and door-to-door campaigns.	
6. Patient Non-Compliance			

## Data Availability

The data that support the findings of this study are available from the corresponding author, S.v.Z., upon reasonable request.

## Appendix A

**Table A1:** Overarching themes, sub-themes, and an overview of the main findings of focus group discussions with primary health care team and patients with chronic diseases of lifestyle (CDLs) [35].

**Main Findings of Focus Group Discussions with Urban and Rural Primary Healthcare Team Members**		
**Overarching Theme**	**Sub-Themes**	**Overview of Main Findings**
Knowledge of CDL protocols/guidelines	Awareness and availability of guidelines Range of CDL covered. Relevance of the guidelines for the SA context.	Urban and rural: ○ CDL protocols and guidelines available aim to achieve the goals of CDL intervention. ○ Guidelines for some risk factors are not available. Urban: ○ CDL protocols and guidelines are consolidated into one book. ○ It covers a range of CDL and is relevant to the SA context. Rural: ○ Use guidelines in clinic and community workers for patient education.
Training to implement guidelines and protocols	Training program. Training needs. Challenges experienced.	Urban: ○ The facilitator provides training at the clinic. ○ Currently, there is no training and updates due to a staff shortage. Rural: ○ Need for recurrent and thorough training.
Practical implementation of CDL protocols and guidelines	Barriers/challenges experienced: Appropriateness of guidelines. Socio-economic conditions. Patient compliance. Resource constraints (human, medication). Accessibility of PHC settings. Infrastructure and security.	Urban and rural: ○ Guidelines are not always practical. ○ Certain CDL risk factors are not covered in the guidelines. ○ The socio-economic conditions of patients/communities are not considered. ○ Non-compliance and/or denial. ○ Lack of health education (staff shortage, time constraints, misinformation). ○ Transport problems. Urban: ○ No support groups. ○ Bedridden patients, side effects of medication. Rural: barriers/challenges ○ Resource constraints: the shortage of stock. ○ Patient referrals and transport over weekends. ○ PHC facility: Infrastructure, security.
Proposed solutions and current best practices	Proposed solutions Fully functional and active PHC teams. Focus on a patient-centred approach. Address human resource constraints. Improve community-based health education strategies.	Urban ○ Holistic patient-centred approach. Patients taking ownership of the disease. ○ Focused health education and awareness campaigns—multi-media approach (TV/videos for patient’s education followed by questions and answer sessions, posters, pamphlets (multi-lingual), newspapers. ○ Active support groups. ○ Functional community outreach teams (community health workers—home visits and door-to-door campaigns) and multidisciplinary teams (important roles of dieticians and phycologists.) Rural: ○ An active community outreach team. Staff appointments. ○ Improve management reporting system.
**Main Findings of Focus Group Discussions with Urban and Rural Patients with Chronic Diseases of Lifestyle**		
**Overarching Themes**	**Sub-Themes**	**Overview of Main Findings**
Knowledge of CDL	Knowledge of factors that contribute to the development of CDL. Knowledge of factors that prevent the development of CDL. Knowledge of the pathophysiology/development of CDL. Long-term treatment of CDL.	Urban and rural: ○ Genetic factors, lifestyle factors, dietary factors, physiological factors, and socio-economic factors. ○ Regular check-ups, taking medication, and monitoring of adverse effects of medication.
Health promotion and education	Factors contributed to participants’ knowledge of CDL. Type of health education and additional health promotion services provided.	Urban and rural: ○ Doctors and nursing staff in the clinic, pamphlets, and radio. Urban: ○ Sometimes nursing staff, dieticians, pamphlets (urban—none at the clinic, mainly in the hospital). Rural: Home visit
Experience of the quality of CDL health care at the primary health care facility	Factors that affect the delivery of optimal, quality health care service at PHC facilities Resource constraints (human, medication) Infrastructure Security Transport issues Lack of supporting health services	Urban and rural: ○ Poor/lack of services (patient load/staff shortage). ○ Lack of supporting health care services. ○ Transport. ○ Need for support groups. ○ Distance to travel to primary healthcare facilities. Urban: ○ Long waiting time. ○ Shortage of medicine. ○ Lack of holistic care. ○ Mobile clinics are not available. ○ Poor service (no physical examinations). Rural: ○ Security ○ Infrastructure (dilapidated clinic building, ablution facilities, and terrain). ○ Shortage of support staff (cleaners).
